# Heavy Metal Pre-Conditioning History Modulates *Spartina patens* Physiological Tolerance along a Salinity Gradient

**DOI:** 10.3390/plants10102072

**Published:** 2021-09-30

**Authors:** João Carreiras, Jesús Alberto Pérez-Romero, Enrique Mateos-Naranjo, Susana Redondo-Gómez, Ana Rita Matos, Isabel Caçador, Bernardo Duarte

**Affiliations:** 1 MARE—Marine and Environmental Sciences Centre, Faculty of Sciences of the University of Lisbon, Campo Grande, 1749-016 Lisbon, Portugal; jgcarreiras@fc.ul.pt; 2 Departamento de Biología Vegetal y Ecología, Facultad de Biología, Universidad de Sevilla, Av. Reina Mercedes s/n, 41012 Sevilla, Spain; jesusalperezromero@gmail.com (J.A.P.-R.); emana@us.es (E.M.-N.); susana@us.es (S.R.-G.); 3 Plant Functional Genomics Group, BioISI—Biosystems and Integrative Sciences Institute, Departamento de Biologia Vegetal, Faculdade de Ciências da Universidade de Lisboa, Campo Grande, 1749-016 Lisboa, Portugal; armatos@fc.ul.pt; 4 Departamento de Biologia Vegetal, Faculdade de Ciências da Universidade de Lisboa, Campo Grande, 1749-016 Lisbon, Portugal; micacador@fc.ul.pt

**Keywords:** halophytes, osmotic stress, pre-conditioning, intraspecific variability

## Abstract

Land salinization, resulting from the ongoing climate change phenomena, is having an increasing impact on coastal ecosystems like salt marshes. Although halophyte species can live and thrive in high salinities, they experience differences in their salt tolerance range, being this a determining factor in the plant distribution and frequency throughout marshes. Furthermore, intraspecific variation to NaCl response is observed in high-ranging halophyte species at a population level. The present study aims to determine if the environmental history, namely heavy metal pre-conditioning, can have a meaningful influence on salinity tolerance mechanisms of *Spartina patens*, a highly disperse grass invader in the Mediterranean marshes. For this purpose, individuals from pristine and heavy metal contaminated marsh populations were exposed to a high-ranging salinity gradient, and their intraspecific biophysical and biochemical feedbacks were analyzed. When comparing the tolerance mechanisms of both populations, *S. patens* from the contaminated marsh appeared to be more resilient and tolerant to salt stress, this was particularly present at the high salinities. Consequently, as the salinity increases in the environment, the heavy metal contaminated marsh may experience a more resilient and better adapted *S. patens* community. Therefore, the heavy metal pre-conditioning of salt mash populations appears to be able to create intraspecific physiological variations at the population level that can have a great influence on marsh plant distribution outcome.

## 1. Introduction

According to the analysis of the data gathered, through this and the last century, the Intergovernmental Panel on Climate Change (IPCC) report shows a worldwide intensification of abiotic stresses with alarming environmental and economic implications, notably the increase and intensity of extreme climate events, droughts, floods, sea-level rise, water, and land salinity variations and others [1]. Earth can be considered a salt planet since approximately, 97.5% of all planet’s water content is saltwater, occupying roughly 70% of the surface encompassed in oceans, lakes, and groundwater [2], furthermore, it has been estimated that high soil salinity is affecting 20% of total Earth’s land surface and 33% of agricultural irrigated lands [3,4].

In coastal regions, especially in the high populated low-elevation coastal lands and estuaries, climate change will likely increase, at an elevated rate, the soil, and water salinity, mostly due to predicted storm surges, tidal surges, and sea-level rise causing an onward saltwater land inundation. Therefore, it can be presumed that soil and water salinity-induced stress is and will be one of the major plant abiotic stresses. Usually, salt stress in plants is a powerful limiting production factor, upsetting every major crop development and productivity [5]. Most of the crop plants when exposed to NaCl concentrations from 40 mM to 200 mM become severely damage or die, plants exposed to elevated salt concentrations result in several complex biochemical, physiological, and morphological damages, such as nutrient uptake and assimilation [6,7,8]. On the other hand, and contrary to 99% of all the plant species, halophytic vegetation species can not only survive but be highly productive in saline environments. Halophytes are, by definition, plants that can live normally and complete their life cycle under a salt concentration of at least 200 mM, with most plants exhibiting tolerance to a remarkable amplitude of NaCl concentration [9]. However, it is known that different halophytes species have unlike responses to the same salinity, ranging from species having optimal performance in salt-free environments to high NaCl concentrations such as 400 mM [9,10]. Species salinity tolerance responses variations are relevant when taking into consideration the latent alterations to salinity in the environment, which will most likely change salt-tolerant plant habitat availability and distribution, within an ecosystem. This is evident in most halophytes inhabiting salt marshes, where species distribution is organized across salinity gradients and microhabitats salinity variations associated with marsh topography and morphology, where plants are arranged according to their salinity tolerance [11,12]. Nonetheless, intraspecies phenological and physiological variation phenomenon can occur to a greater degree in highly tolerant species that are capable to adapt to environments that largely differ in their abiotic conditions [13,14,15]. When intraspecific NaCl response is taken into consideration it may show a different response to the same NaCl concentration, therefore it is important to understand coastal ecosystem modification and evolution once exposed to salinity changes.

Tagus estuary wetland is considered one of the more important in Europe and encompasses the most extensive and continuous salt marsh area in Portugal, presenting a great concentration of organic matter and biological productivity [12]. Salt marshes located within this estuary share most of the colonized halophyte species, such as the halophyte *Spartina patens* (Aiton) Muhl, a highly tolerant, invasive salt-excreting grass that is now spreading across Mediterranean marshes [16]. Moreover, neighboring marshes within the Tagus estuary, although being under mostly similar abiotic conditions, like salinity and temperature, can, due to anthropogenic actions, display a significant difference in their soil chemistry, when comparing marshes located within natural reserves to industrially contaminated marshes, notably caused heavy metals pollution [17]. These aspects make this species a suitable model to understand the effects of metal pollution pre-conditioning on tolerance range to salinity variations and ascertain to what extent different populations could otherwise respond to future changes in soil and water salinity. Additionally, several studies have suggested that intraspecific salt tolerance variations can occur in different *S. patens* populations [18,19], as well as heavy metal pre-conditioning can have a significant impact on this plant abiotic tolerance mechanisms [20].

The present work intends to determine heavy metal cross-tolerance thru pre-conditioning to salinity stresses in *S. patens*. Employing imposing salinity treatments on two populations, one from a heavy metal contaminated marsh and the other from a pristine one, it was possible to evaluate significant intraspecific variability in the physiological salt tolerance mechanisms. Given the ongoing climate change, it is relevant knowledge of the differently salt marshes species potential to adapt and respond, as well as the perception of a more complex reaction directly related to future salt-induced habitat modifications, concerning plant distribution and frequency.

## 2. Results

### 2.1. Photochemical Processes

**Figure 1:**
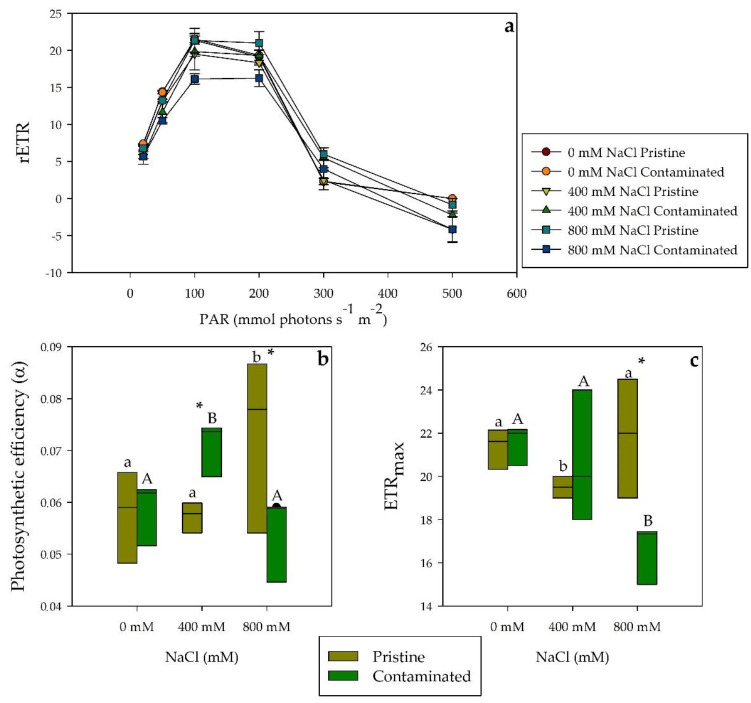
(**a**) Relative electron transport rates (rETR), (**b**) photosynthetic efficiency (α), and (**c**) maximum electron transport rate (ETR_max_) in *S. patens* dark-adapted leaves from pristine and heavy metal contaminated sites (average ± standard error, N = 5), along with the tested NaCl concentrations. Letters indicate significant differences between treatments at *p* < 0.05; asterisks mark significant differences between populations at *p* < 0.05.

When exposed to a salinity gradient, *S. patens* showed substantial variances in terms of their photochemical responses at a population level. The relative electron transport rates (rETR; Figure 1a) variation was shown to be significantly different, between populations, at 800 mM NaCl, higher values in the pristine marsh population. The photosynthetic efficiency (α; Figure 1b), measured within populations, displayed stability along the applied salinity gradient, with only individuals from the heavy metal contaminated marsh showing a significant difference at 400 mM NaCl. Nevertheless, significant differences were found between populations, contaminated marsh individuals displayed higher photosynthetic efficiency at 400 mM NaCl however at 800 mM NaCl the opposite was found. Maximum electron transport rates (ETR_max_; Figure 1c) measurements showed a significant variation in 800 mM NaCl conditions between the populations, lower in the contaminated site individuals.

**Figure 2:**
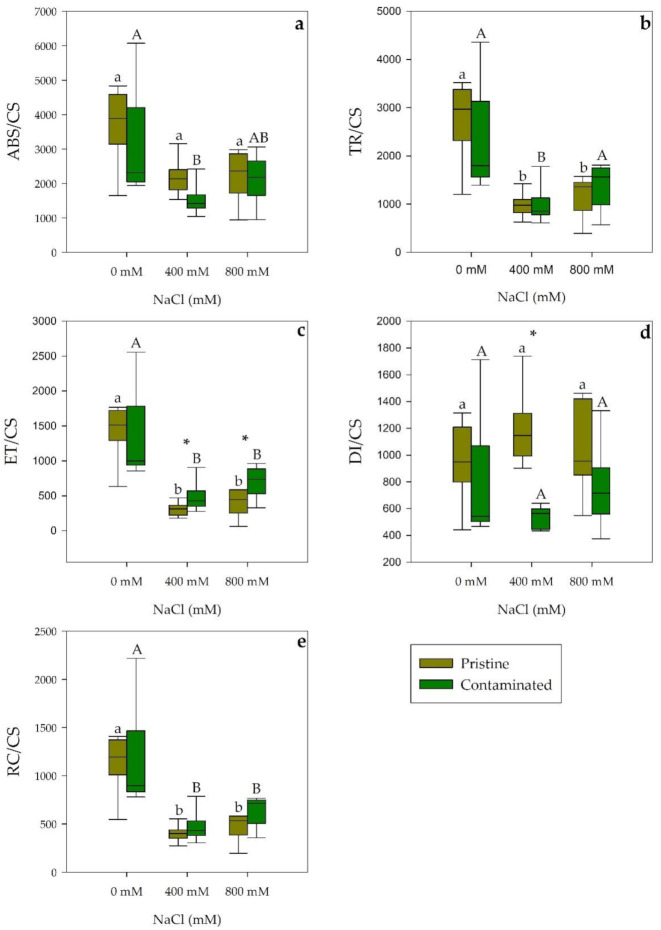
Phenomenological energetic parameters, (**a**) absorbed energy flux (ABS/CS), (**b**) trapped energy flux (TR/CS), (**c**) electron transport energy flux (ET/CS), (**d**) dissipation energy flux (DI/CS), and (**e**) oxidized reaction centers (RC/CS) on a cross-section basis, in *S. patens* dark-adapted leaves from pristine and heavy metal contaminated sites (average ± standard error, N = 5), along with the tested NaCl concentrations. Letters indicate significant differences between treatments at *p* < 0.05; asterisks mark significant differences between populations at *p* < 0.05.

Energetic fluxes per leaf cross-section of the salt-treated chloroplasts showed a decrease in both populations under saline conditions in absorbed energy flux along a salinity gradient (ABS/CS; Figure 2a), significant in the 400 mM NaCl treated samples from the contaminated marsh. In trapped energy flux (TR/CS; Figure 2b) a similar significant reduction was observed in both populations although this decrease was not significant in the population from the heavy metal contaminated location at 800 mM NaCl, possibly due to the comparatively lower values exhibited in the 0 mM NaCl exposed individuals. Electron transport energy flux (ET/CS; Figure 2c) displayed a significant reduction at 400 and 800 mM NaCl, being this reduction more acute in *S. patens* from the pristine site. Dissipation energy flux (DI/CS; Figure 2d) showed unlike and significant responses to salinity stress between salt marsh populations. Plants from the pristine site when exposed to 400 mM NaCl showed increase energy dissipation whilst the contaminated site individuals exposed exhibited a reduction in dissipation. Finally, oxidized reaction centers (RC/CS; Figure 2e) significantly decreased with salinity concentration to a similar degree in both sampling populations.

**Figure 3:**
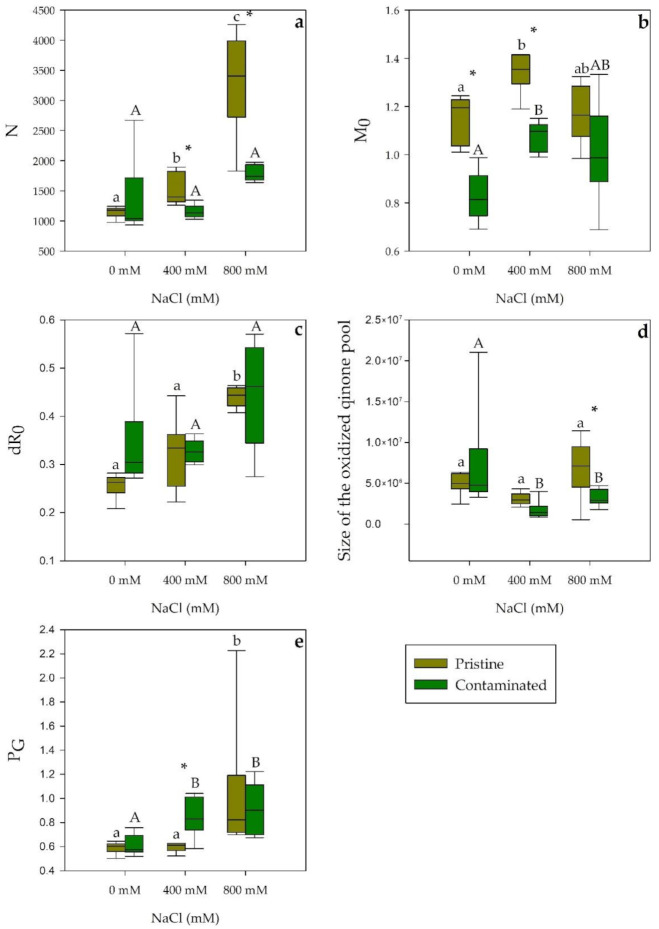
OJIP derived parameters, (**a**) the total number of electrons transferred into the electron transport chain (N), (**b**) the net rate of PS II reaction centers closure (M_0_), (**c**) PS I efficiency in reducing its electron acceptors (δR_0_), (**d**) size of the oxidized quinone pool, and (**e**) grouping probability (P_G_) in *S. patens* dark-adapted leaves from pristine and heavy metal contaminated sites (average ± standard error, N = 5), along with the tested NaCl concentrations. Letters indicate significant differences between treatments at *p* < 0.05; asterisks mark significant differences between populations at *p* < 0.05.

Considering the total number of electrons transferred into the electron transport chain (N; Figure 3a), contaminated marsh individuals, when salt exposed, exhibited no significant changes while pristine site plants showed an increasing trend, displaying a significant increase at 400 mM NaCl and a highly significant increase at 800 mM NaCl, in relation to the contaminated site population as well at both salinities. Regarding the net rate of PS II reaction centers closure (M_0_; Figure 3b), a significantly higher value was evident in the 400 mM salt treatments of both populations, nonetheless contaminated site samples showed significantly lower M_0_ at 0 and 400 mM NaCl. Electron movement efficiency from the reduced intersystem electron acceptors to the PS I end electron movement (δR_0_; Figure 3c) showed an increase through salinity treatments in *S. patens* from the pristine mash, significant at 400 mM NaCl. The oxidized quinone pool size (Figure 3d) showed a similar pattern when comparing populations, the only difference was exhibited at 800 mM NaCl, a significantly lower size in the contaminated marsh population. Considering the grouping probability (P_G_; Figure 3e), a significantly higher PS II antennae connectivity was exhibited at 800 mM NaCl in both site samples, while at 400 mM NaCl *S. patens* from the contaminated marsh, showed a significantly higher value within and between populations. 

### 2.2. Photosynthetic Pigments Profile

**Figure 4:**
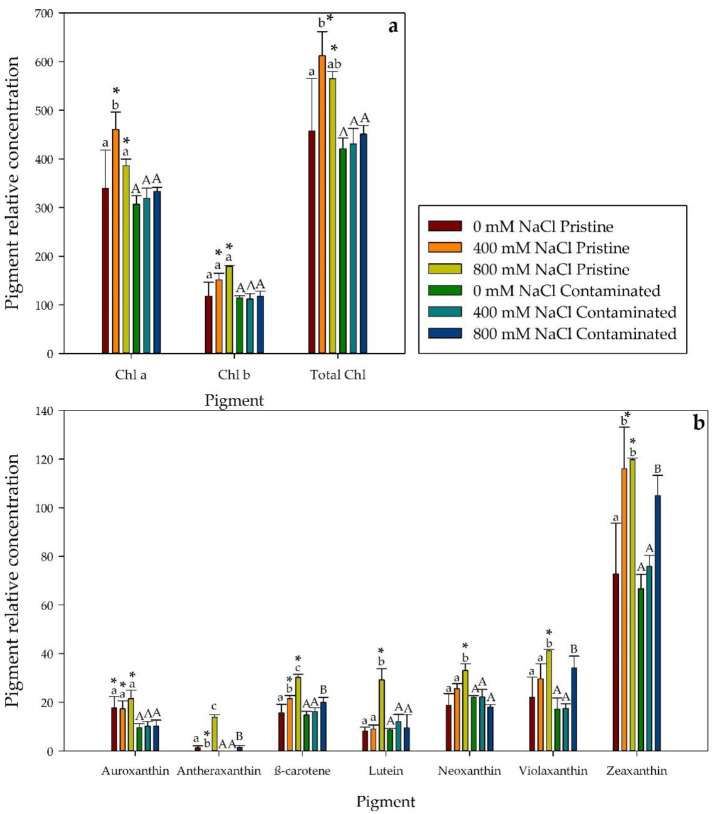
Pigment relative concentration. (**a**) Leaf chlorophyll *a* (Chl *a*), chlorophyll *b* (Chl *b*), total chlorophyll (Total Chl), (**b**) auroxanthin, antheraxanthin, β-carotene, lutein, neoxanthin, violaxanthin and zeaxanthin concentration (µg g^−1^ FW) in *S. patens* individuals from pristine and heavy metal contaminated sites (average ± standard error, N = 5), along with the tested NaCl concentrations. Letters indicate significant differences between treatments at *p* < 0.05; asterisks mark significant differences between populations at *p* < 0.05.

**Figure 5:**
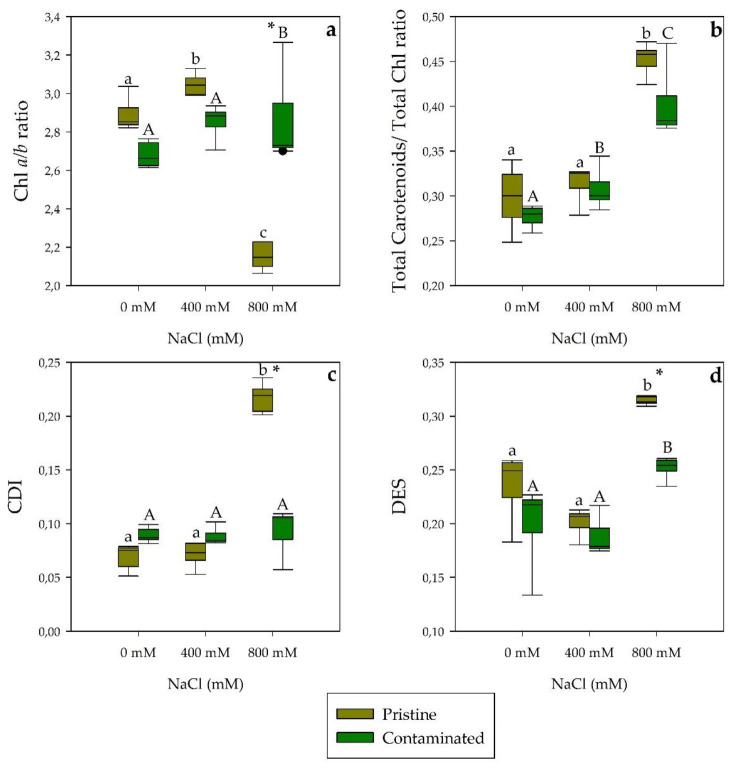
Leaves pigment ratios, (**a**) chlorophyll *a*/*b* ratio (Chl *a/b* ratio), (**b**) total carotenoid to total chlorophyll ratio, (**c**) chlorophyll degradation index (CDI), and (**d**) de-epoxidation state (DES) in *S. patens* individuals from pristine and heavy metal contaminated sites (average ± standard error, N = 5), along with the tested NaCl concentrations. Letters indicate significant differences between treatments at *p* < 0.05; asterisks mark significant differences between populations at *p* < 0.05.

Regarding leaf pigments concentration, we found significant differences between salinity treatments and populations (Figure 4). Thus, total chlorophyll concentration (chl *a* and chl *b*) was significantly higher in the salt-treated pristine marsh individuals compared with their contaminated marsh counterparts (Figure 4a). In addition, in the pristine marsh *S. patens*, higher pigment concentrations were also found, with significance, in auroxanthin in all treatments, in lutein, neoxanthin, and violaxanthin at 800 mM NaCl and β-carotene and zeaxanthin when exposed to 400 and 800 mM NaCl concentrations (Figure 4b). The total carotenoid to total chlorophyll ratio (Figure 5b) displayed a similar pattern, with no significant differences, between populations. Contaminated site samples exhibited a significantly higher Chlorophyll *a*/*b* ratio than the pristine marsh population at 800 mM NaCl (Figure 5a). Furthermore, at 800 mM NaCl, the contaminated marsh *S. patens* showed a significantly lower chlorophyll degradation index (CDI, Figure 5c) and de-epoxidation state (DES, Figure 5d).

### 2.3. Antioxidant Enzymatic Activities

**Figure 6:**
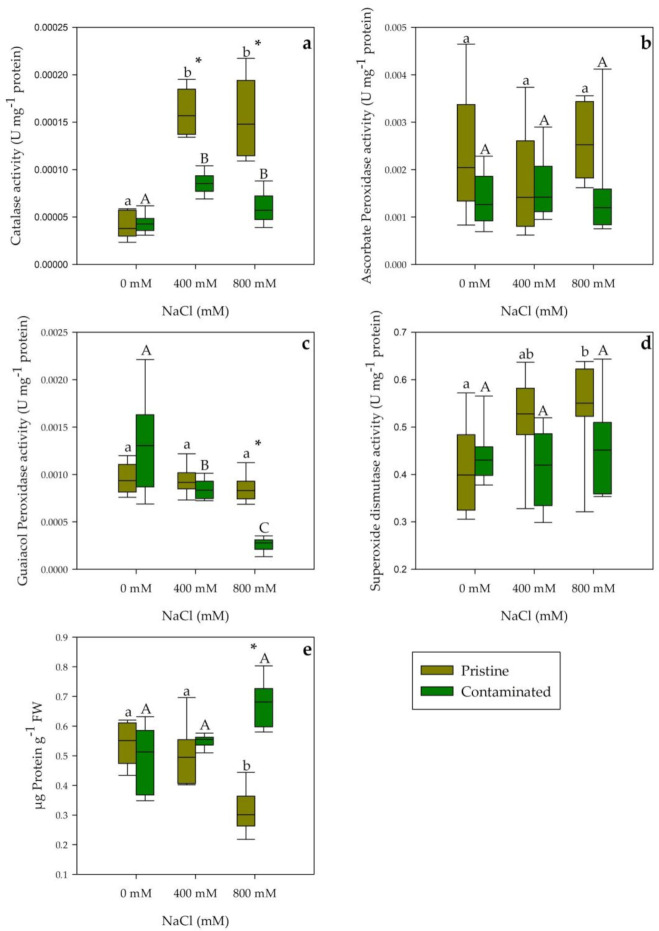
(**a**) Catalase, (**b**) ascorbate peroxidase, (**c**) guaiacol peroxidase, and (**d**) superoxide dismutase activities (U mg^−1^ protein) and (**e**) total protein content (µg Protein g^−1^ FW) in the leaves of *S. patens* from pristine and heavy metal contaminated sites (average ± standard error, N = 5), along with the tested NaCl concentrations. Letters indicate significant differences between treatments at *p* < 0.05; asterisks mark significant differences between populations at *p* < 0.05.

Catalase activity presented a highly significant increase in salt treatments, being these values considerably higher in plants from the pristine marsh (Figure 6a). Contrarily ascorbate peroxidase activity and superoxide dismutase activity did not show any significant variations between both tested populations and salinity concentrations (Figure 6b,d). Guaiacol peroxidase activity showed a significant activity decrease through NaCl concentration gradient in individuals from the contaminated marsh, while its values did not vary with salinity concentration in those collected in the pristine site (Figure 6c). Finally, regarding the total protein content of the leaves, a decreasing tendency was observed in the pristine site samples, significant at 800 mM NaCl within and between population groups (Figure 6e).

### 2.4. Fatty Acid Composition

**Figure 7:**
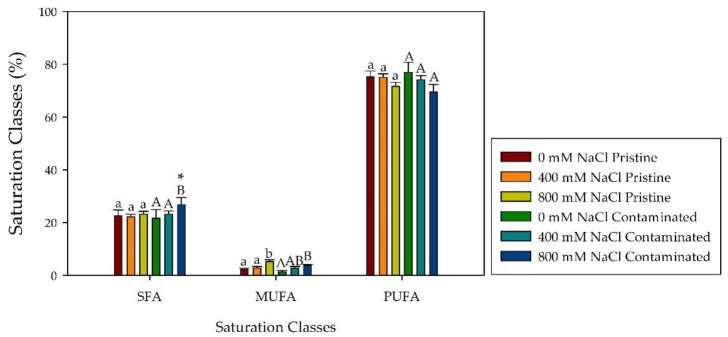
Saturated fatty acid (SFA), monounsaturated fatty acid (MUFA) and polyunsaturated fatty acid (PUFA) relative concentration (%, average ± standard error, N = 5) in *S. patens* leaves from pristine and heavy metal contaminated sites, along with the tested NaCl concentrations. Letters indicate significant differences between treatments at *p* < 0.05; asterisks mark significant differences between populations at *p* < 0.05.

**Figure 8:**
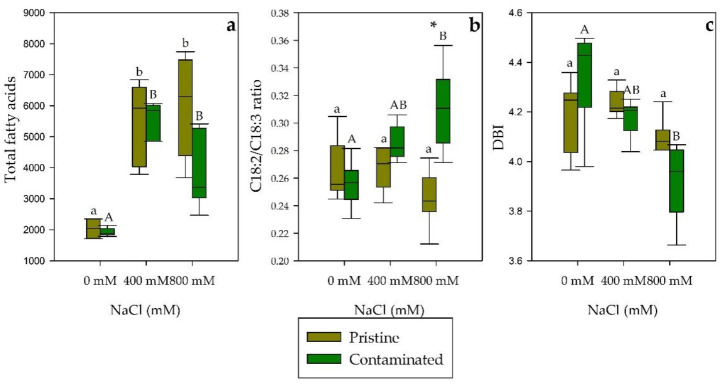
(**a**) Total fatty acid content (µg.g^−1^ FW), (**b**) linoleic acid to linolenic acid ratio (C18:2/C18:3 ratio), and (**c**) double-bound index (DBI) in *S. patens* leaves from pristine and heavy metal contaminated sites (average ± standard error, N = 5), along with the tested NaCl concentrations. Letters indicate significant differences between treatments at *p* < 0.05; asterisks mark significant differences between populations at *p* < 0.05.

Regarding fatty acid leaf content under salinity exposure (Table 1), the most abundant fatty acids found in the tested groups were palmitic acid (C16:0), linoleic acid (C18:2), and linolenic acid (C18:3). *Spartina patens* individuals from the pristine marsh when exposed to salinity showed an increase in palmitic acid, while the individuals from the contaminated site showed a decrease, displaying a significantly different trend between populations. An opposite trend, between the *S. patens* populations, was also found in the stearic acid (C18:0) concentration, increasing and decreasing through salinity treatments in the individuals from the contaminated and pristine marsh respectively. Trans-delta 3-hexadecenoic acid (C16:1t) displayed a significantly higher percentage in the pristine marsh group in 0 mM and 800 mM NaCl. Both populations displayed a decrease in linolenic acid content as a result of NaCl treatments. Considering the fatty acid saturation classes in salt-treated leaves, both marsh populations displayed similar trends. However, saturated fatty acid (SFA) at 800 mM NaCl was found to be significantly higher in the contaminated site samples (Figure 7). The total fatty acid content of *S. patens* presents highly significant increases in both population treatments (Figure 8a). Contaminated site individuals, when exposed to increasing salinities, displayed an increasing trend in the C18:2/C18:3 ratio (Figure 8b), as well as an inverse trend in the double bond index (DBI; Figure 8c). In contrast, in NaCl treated plants from the pristine site no significant changes were observed.

### 2.5. Multivariate Classification

**Figure 9:**
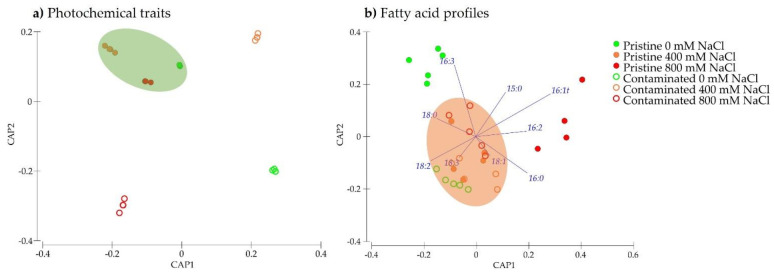
Canonical analysis of principal coordinates (CAP) based on (**a**) photochemical traits and (**b**) based on the fatty acid profiles, pentadecanoic acid (C15:0), palmitic acid (C16:0), trans-delta 3-hexadecenoic acid (C16:1t), hexadecatrienoic acid (C16:3), stearic acid (C18:0), oleic acid (C18:1), linoleic acid (C18:2) and linolenic acid (C18:3)) from *S. patens* collected in pristine and heavy metal contaminated salt marshes exposed to the tested NaCl concentrations.

Gathering all the photochemical data (full Kautsky induction curve dataset) into a unifying canonical analysis of principal coordinates (CAP) the abovementioned differences and traits are highlighted in an integrative form. Moreover, the cross-validation step of this canonical analysis presented a highly elevated classification efficiency of more than 95% for allocation within groups, reinforcing the statical differences observed at the individual level of each of the photochemical traits as efficient descriptors of the populations’ behavior along the tested salinity gradient (Figure 9a). The pristine marsh individuals were grouped and identified, sharing similar photochemical traits, while the individuals from the contaminated site evidence a clear separation under the exposure to different salinity values. A similar approach was performed regarding the leaf fatty acid profile, with the CAP projection based on these traits producing a different grouping profile. Intermediate salinity exposed samples from both populations shoed similar fatty acid profiles being grouped in the center of the projection alongside the samples from individuals collected at the contaminated site exposed to 0 mM NaCl (Figure 9b). Samples from the pristine site exposed to the lowest and highest salinity treatment tested were grouped differentially from the remaining samples. Worth noticing that the CAP analysis based on the fatty acid analysis showed a lower classification efficiency (approximately 70%). Both these CAP analyses show to highlight the different impacts of the tested salinity treatments in the photochemical and fatty acid metabolism, and the different feedbacks from each of the *S. patens* populations. 

## 3. Discussion

Effects of environmental change on coastal regions include the progressive land immersion from ocean level rise, heightened storm damage, expanding drought seasons, and temperature increase [21]. These abiotic alterations reveal to have significant implications on the environmental salinity gradient, with recent studies forecasting disturbing impacts in salinity concerning waterfront areas [22,23,24]. Therefore, salt marshes ecosystems will be largely affected, especially when considering the salinity concentration to be one of the major constraints of species frequency, distribution, and zonation along with the marsh profile [25,26]. However, complex and significant interspecific variations in the salinity responses of halophytes due to pre-conditioned histories can be a factor in the adaptation of neighboring marsh populations [20].

Photochemical analysis of *S. patens*, when exposed to salt treatments, confirmed that this species has a high degree of tolerance to salinity even at high NaCl concentrations as shown in previous studies [27,28]. Nevertheless, noticeable differences were shown between NaCl treatments as well as between pristine and contaminated marsh populations. Considering the electron transport chain and related parameters in salt-treated *S. patens*, it was observed a significant intraspecific difference at 400 and 800 mM NaCl. The individuals from the contaminated marsh, at 400 mM NaCl, displayed a significantly higher photosynthetic light efficiency, coupled with relatively low dissipation energy (DI/CS), which suggests a high electronic transport chain efficiency [20]. On the other hand, at 800 mM NaCl, contaminated marsh samples show a significantly lower maximum ETR and photosynthetic efficiency suggesting an inferior electron transport chain proficiency at higher salinity concentration when compared to the heavy metal contaminated individuals. According to the data acquired, in the salt exposed groups, there is a linear relationship between the variation found in the oxidized quinone pool size and the electron transport energy flux (ET/CS), the absorbed energy flux (ABS/CS), the trapped energy flux (TR/CS), as well as the available reaction centers (RC/CS), nonetheless, the populations showed significant differences among these parameters. Even though the contaminated site *S. patens*, at 400 and 800 mM NaCl, when compared to the pristine site samples, showed a more significant decrease in quinone pool size, the reduction in the electron transport energy flux was less significant, this can be explained by a better PS II efficiency associated with the lower dissipation energy flux found in the individuals from the contaminated marsh, especially at 400 mM NaCl was the intraspecific differences were found to be more significant [29]. Although the size of the oxidized quinone pool, in the individuals from the pristine location, displayed no significant changes when subjected to salinity, the number of quinone turnovers increased, showing lower quinone pool reduction rates [30]. In contrast, the individuals from the contaminated marsh exhibited a significant reduction of the quinone pool size and no significant changes in its turnover time, indicating tolerance mechanisms that allowed the maintenance of electronic flow rate from the reduced quinone pool to the electron transport chain [10]. 

The Xanthophyll cycle is a well-described mechanism of energy dissipation, commonly observed in halophytic plants [10,30,31]. To reduce energy overload within light-harvesting complexes (LHCs), the de-epoxidation of the violaxanthin pool towards the zeaxanthin is normally activated [32]. *Spartina patens* when exposed to salinity, only in 800 mM NaCl treatments in both population samples, showed a highly significant increase in de-epoxidation, reflection of a higher activity of the xanthophyll cycle attempting to scatter the excessive redox potential amassed inside the stroma. The activity shift to photoprotection in the higher salinities was also clear due to the significant rise of the total carotenoid to total chlorophyll ratio. As a possible countermeasure against reactive oxygen species [31] significant increases were observed in β-carotene and lutein, antioxidant acting, pigment concentrations in the salt exposed populations, in particular, this phenomenon was found with more significance at the higher salt concentration in the individuals from the pristine marsh. A highly significant increase in β-carotene was present in the contaminated site samples at 800 mM NaCl and both salinity treatment in the plants from the pristine location, as well as a highly significant increase in lutein at 800 mM NaCl. This may indicate a better ROS savaging capability by *S. patens* from the contaminated marsh. 

The interaction of high NaCl concentrations with the cell organelles leads to increased production of ROS resulting in potentially harmful physiological reactions within the plant cells, affecting among others, proteins production and metabolism [33,34]. Halophytes built up a highly proficient system of enzymatic rapid responses toward salinity changes, immediately activated when the environmental conditions shift aside from the saline comfort zone [35]. When assessing the oxidative stress biomarkers in *S. patens*, discrepancies in the responses to the salinity stress between populations are clear. Contrary to what was found in the contaminated marsh plants, the pristine site individuals displayed an increase of antioxidant enzyme activities, revealed by the increase of superoxide dismutase and catalase activity when salt treated, significant at 800 mM NaCl [36]. This, coupled with a decrease in total protein content found in this same group, suggests that, when exposed to 800 mM NaCl, *S. patens* from the pristine marsh, when comparing to the contaminated marsh population, as a higher ROS production, as well as comparatively inferior scavenging mechanism of the ROS species [37,38]. 

The fatty acid profiles of the salt exposed halophytes presented similar responses amongst populations, but some differences are noteworthy. The linoleic (C18:2) and linolenic (C18:3) ratios, considered a salt stress indication, when under stress conditions the ratio shifts towards linolenic, since it is a membrane restructuring with lower amounts of polyunsaturated acids, thus inferior C18:3 concentration in the leaf is considered an adaptation to salt exposure [39]. The C18:2/ C18:3 ratio increased exclusively in the salinity treated *S. patens* from the contaminated site, therefore it can be suggested that these individuals are less stressed than those from the pristine location. Furthermore, C18:3 can also act as a direct non-enzymatic reactive oxygen species scavenger [40], which complies with, comparatively, lower ROS consequences found previously in the *S. patens* from the contaminated marsh. Furthermore, the population from the contaminated marsh displayed a highly significant rise in oleic acid (C18:1), known for improving the stabilization of light-harvesting complexes [41], seen by the positive significant correlation between LHC stress indicator chl *a*/ chl *b* ration and the C18:1 fatty acid significant correlation (r^2^ = 0.921; *p* < 0.05). On the other hand, in the individuals from the pristine site, the correlation between these two variables is quite low (r^2^ = 0.161; *p* < 0.05), indicating that this mechanism only occurs in the plants from the contaminated marsh. Tras-delta-3 hexadecenoic acid (C16:1t), exclusive to plastids [42] and the only strictly light-dependent fatty acid, enables the correct organization of light-harvesting antennae complexes [30,43,44,45]. When comparing the individuals subjected to 0 mM NaCl from both populations, a significant increasing trend was found in the C16:1t concentration of the individuals from the contaminated marsh, concomitant with the, previously determined, lower energy dissipation and reduced reaction centers turnover and closure rates found in the plants from the contaminated marsh, comparatively to those found in the individuals from the pristine marsh, proposing a better LHC organization and heath in the heavy metal affected *S. patens* when exposed to salt stress.

The overall physiological shift was observed in the CAP analysis where it was compared the physiological and photochemical variations of the individuals under the different NaCl treatments. The cross-validation provided an efficient approach to classify and assess the changes and effects in both populations [46]. When observing the multivariate analysis, NaCl treated *S. patens* from the pristine marsh showed a clear grouping at the photochemical changes, however, when using the fatty acids profile as the basis the grouping was seen in the contaminated marsh populations. This distinct classification efficiently displays *S. patens* intraspecific variation. The higher degree of efficiency in the classification of the samples observed in the photochemical traits-based CAP analysis indicates that not only this metabolism is more affected (thus producing more pronounced differences between sample groups) but also that has a higher ability to be used as biomarkers in similar studies comparing not only salinity treatments but also plant populations. Although fatty acid profiles are known to be sensitive to osmotic stress in this particular species as well as in other halophyte species when comparing the same species along a salinity gradient [28,47,48], this canonical approach loses sensitivity when comparing populations of the same species exposed to the same salinity treatments, pointing out to a prevalent role of the salinity treatment over the population origin, in this case, thus leading to less efficient fatty acid-based canonical analysis.

## 4. Material and Methods

### 4.1. Sampling Sites and Plant Material Collection

**Figure 10:**
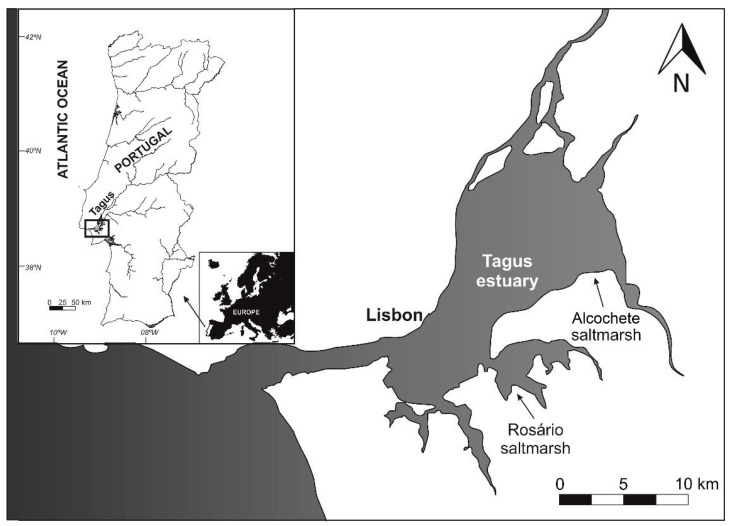
Tagus Estuary map with Alcochete (pristine) and Rosário (heavy metal contaminated) salt marshes sampling stations marked [50].

Sampling was carried out on the Tagus estuary, located in the western coast of Portugal, one of the larger estuaries in occidental Europe with an area of approximately 320 km^2^ (38°44′ N, 9°03′ W; Figure 10). The estuary involves a watershed superior to 80,000 km^2^ in Spain and Portugal territories, being the second most significant hydrological basin in the whole Iberian Peninsula.

*Spartina patens* sampling was done during low tide in the southern part of the Tagus estuary in September 2017, on the same day and tidal period at two sampling sites: Alcochete salt marsh (38°45′ N, 8°56′ W) situated within the Tagus Estuary Natural Reserve and Rosário salt marsh (38°40′ N, 9°01′ W) in the vicinity of a former industrial area (Figure 10). Whole plants were excavated from the sediment and intact individuals were transported individually to the laboratory (in refrigerated bags and quickly transported (less than an hour) to the laboratory. Due to the high proximity between both sampling sites, the plant phenological cycle is not different, with very similar plants in terms of morphology and biomass between both sites (data not shown). The geographical location of both marshes prompts a differential metal contamination exposure from anthropogenic origins [28,31]. This is reflected in the bioavailable metal concentrations found in both marshes, with Alcochete sediments showing non-detectable bioavailable Cd concentrations, 0.023 ppm of Cu, 0.001 ppm of Ni, 0.022 ppm of Pb, and 0.052 ppm of Zn on average [31]. On the other hand, Rosário salt marsh sediments exhibited much higher bioavailable metals average concentration values, presenting 0.001 ppm of Cd, 0.034 ppm of Cu, 0.003 ppm of Ni, 0.116 ppm of Pb, and 0.233 ppm of Zn [20,33]. Considering these values, Alcochete marsh was classified as pristine and Rosário marsh as heavy metal contaminated.

At the laboratory, plant samples were gently washed to remove dust and sediments. *Spartina patens* intact tussocks were set in pots (*N* = 5) filled with perlite and irrigated with ¼ Hoagland nutrient solution [49]. For experimental proposes, individuals were chosen to have all experimental units with individuals presenting similar height and apparent biomass (data not shown). Plants were placed in a phytoclimatic chamber programmed to simulate a natural light environment using a sinusoidal function (maximum PAR 300 µmol photons m^2^ s^−1^, 16/8 h day/night rhythm, 20/18 °C day/night temperature amplitude, relative humidity, 50 ± 2%), and kept under these conditions for 2 months to acclimate to the new growth conditions.

### 4.2. Experimental Setup

After the abovementioned adaptation period, *S. patens* individuals from both sites (pristine and contaminated) were separated into 3 groups with 5 replicate individuals (pots). The sample groups were placed in a phytoclimatic chamber programmed to simulate a natural light environment using a sin function (maximum PAR 300 µmol photons m^2^ s^−1^, 16/8 h day/night rhythm, 20/18 °C day/night temperature amplitude, relative humidity, 50 ± 2%) and the Hoagland nutrient replaced, in two sample groups, with salinity treatment solution of ¼ Hoagland solution supplemented with NaCl to attain the desired target salinities (400 and 800 mM). Exposure trials lasted for 7 days after which chlorophyll fluorescence measurements were made and consecutively, plants were harvested. Leaf samples for biochemical measurements were immediately flash-frozen in liquid-N_2_ and stored at −80 °C until analysis. 

### 4.3. Pulse Amplitude Modulated (PAM) Fluorometry

Modulated chlorophyll fluorescence measurements were made in attached leaves with a FluoroPen FP100 PAM (Photo System Instruments, Czech Republic). All the measurements in the dark-adapted state were made after the darkening of the leaves for at least 30 min. Rapid light curves (RLC) measurements, in dark-adapted leaves, were attained using the preprogrammed LC1 protocol of the FluorPen, consisting of a sequence of pulses from 0 to 500 µmol m^−2^ s^−1^. Each ΦPS II measurement was used to calculate the electron transport rate (ETR) through photosystem II using the following equation: ETR = ΦPS II × PAR × 0.5, where PAR is the actinic photosynthetically active radiation generated by the FluoroPen and 0.5 assumes that the photons absorbed are equally partitioned between PS II and PSI [51]. Without knowledge of the actual amount of light being absorbed, fluorescence measurements can only be used as an approximation for electron transport [52,53,54]. Rapid light curves (RLC) were generated from the calculated ETRs versus irradiance applied plot and fitted to a double exponential decay function to quantify the characteristic parameters, alpha and ETR_max_ [55,56]. The OJIP transient (or Kautsky curves) depicts the rate of reduction kinetics of various components of PS II. This is obtained when a dark-adapted leaf is illuminated with the saturating light intensity of 3500 µmol m^−2^ s^−1^ then it exhibits a polyphasic rise in fluorescence (OJIP): level O represents all the open reaction centers at the onset of illumination with no reduction of Q_A_ (fluorescence intensity lasts for 10 ms); O to J transient indicates the net photochemical reduction of Q_A_ (the stable primary electron acceptor of PS II) to Q_A_^−^ (lasts for 2 ms); the J to I transition is due to all reduced states of closed RCs such as Q_A_^−^ Q_B_^−^, Q_A_ Q_B_^2−^ and Q_A_^−^ Q_B_ H_2_ (lasts for 2–30 ms); P-step coincides with a maximum concentration of Q_A_^−^ Q_B_^2^ with plastoquinol pool maximally reduced and also reflects a balance between the light incident at the PS II side and the rate of utilization of the chemical (potential) energy and the rate of heat dissipation [57]. Table 2 summarizes all the parameters that could be calculated from the fluorometric analysis.

### 4.4. Pigment Profiling

Ground freeze-dried leaf samples were extracted with 100% acetone added and subjected to an ultra-sound bath for 1 min to ensure complete disaggregation of the leaf material. Extraction occurred in the dark for 24 h at −20 °C, after which the samples were centrifuged at 4000× *g* at 4 °C for 15 min. Supernatants were scanned from 350 nm to 750 nm in 1 nm steps, using a dual-beam spectrophotometer (Shimadzu UV/VIS UV1601 Spectrophotometer). Finally, the detected pigment sample absorption spectra were analyzed and quantified employing Gauss-Peak Spectra (GPS) method [58]. The sample spectrum was analyzed, through the GPS fitting library, using SigmaPlot Software. This method is based on the sample spectrum fitting, by a linear combination, to the Gauss-peak spectra, that describes each pigment in the detected spectrum, identifying the samples pigment profile, chlorophyll *a*, chlorophyll *b*, auroxanthin, antheraxanthin, β-carotene, lutein, violaxanthin, and zeaxanthin.

For a better evaluation of the light-harvesting and photoprotection mechanisms, the De-Epoxidation State (DES) was calculated as:

(1) $$\mathrm{DES}=\frac{\left(\left[\mathrm{Antheraxanthin}\right]\;+\;\left[\mathrm{Zeaxanthin}\right]\right)}{\left(\left[\mathrm{Violaxanthin}\right]\;+\;\left[\mathrm{Antheraxanthin}\right]\;+\;\left[\mathrm{Zeaxanthin}\right]\right)}$$

### 4.5. Leaf Fatty Acid Composition

Leaf fatty acid analyses were performed by direct trans-esterification of leaf samples as previously described [20,59,60]. Fatty acid methyl esters (FAME) were prepared in glass tubes containing the internal standard heptadecanoate (C17:0), methanol, and sulphuric acid, at 70 °C for one hour. After cooling down the FAME were extracted by adding petroleum and water, vortexed, centrifuged at 4000× *g* for 5 min. The upper layer was dried under a nitrogen stream in a water bath set to 37 °C. After evaporation, 50 µL of hexane was added to the residue and one µL of the solution separated in a gas chromatograph (Varian 3900, Palo Alto, CA, USA) equipped with a hydrogen flame-ionization detector using a fused silica 0.25 mm i.d. × 50 m capillary column (WCOT Fused Silica, CP-Sil 88 for FAME; Varian). The double-bound index (DBI) was calculated using the equation:

(2) $$\mathrm{DBI}=\frac{2\times \;\left(\left(16:1t+18:1\right)+\;2\times 18:2+3\times \;\left(18:3+16:3\right)\right)}{100}$$

### 4.6. Oxidative Stress Biomarkers

For enzyme extractions of *S. patens* leaf samples were retrieved from −80°C storage and extractions were performed according to Tiryakioglu et al. [61], at 4°C. Frozen leaves were homogenized in 50 mM sodium phosphate buffer (pH 7.6) supplemented with 0.1 mM Na-EDTA in a ceramic mortar with a proportion of 500 mg (FW) to 8 mL respectively. The homogenate was centrifuged at 8890× *g* for 20 minutes at 4 °C, and the supernatant was transferred to a test tube and used for the antioxidant enzyme analyses. 

The enzyme activity measurements of catalase (CAT, EC.1.11.1.6.), Ascorbate peroxidase (APx, E.C. 1.11.1.11), Guaiacol peroxidase (GPX, E.C. 1.11.1.7), and Superoxide dismutase (SOD, E.C. 1.15.1.1) were performed in a dual-beam spectrophotometer (Shimadzu UV/VIS UV1601 Spectrophotometer) using quartz cuvettes. Catalase activity assays were performed according to the method of Teranishi et al. [62], by monitoring the H_2_O_2_ consumption and consequent decrease in absorbance at 240 nm (molar extinction coefficient of 39.4 mM^−1^ cm^−1^). Ascorbate peroxidase was measured according to Tiryakioglu et al. [61], by observing the ascorbate oxidation and consequent absorbance reduction at 290 nm (molar extinction coefficient of 2.8 mM^−1^ cm^−1^). Guaiacol peroxidase measurement was performed according to Bergmeyer et al. [63], by monitoring guaiacol oxidation products formation and its increase in absorbance during 60 seconds at 470 nm (molar extinction coefficient of 26.6 mM^−1^ cm^−1^). Superoxide dismutase total activity was assayed according to the method of Marklund and Marklund [64], by measuring the oxidation rate of pyrogallol monitored at 325 nm. The autoxidation of pyrogallol was read without enzymatic extract during the same period and time interval for comparison enabling. Protein quantification was determined using the Bradford method [65].

Membrane lipid peroxidation quantification was performed in *S. patens* leaf samples according to Heath & Packer [66]. First, leaf samples were homogenized in a freshly prepared Thiobarbituric acid (TBA) solution (0.5% (*w*/*v*) TBA in 20% (*w*/*v*) Trichloroacetic acid), in a proportion of 100 mg FW to 2 mL of solution. The homogenate was incubated for 30 min at 95 °C, cooled on ice to stop the reaction, and centrifuged at 4000× *g* for 5 min at 4 °C. The absorbance was read at 532 nm and 600 nm in a Shimadzu UV-1601 spectrophotometer. Malondialdehyde (MDA) concentration was calculated using the molar extinction coefficient, 155 mM^−1^ cm^−1^ when applying the following equation:

(3) $$A_{532nm}-A_{600nm}=\;\left[\mathrm{MDA}\right]mM\times \varepsilon \mathrm{MDA}$$

### 4.7. Statistical Analysis

Statistical analysis of the data derived from the previous analysis was made based on non-parametric tests, due to a lack of normality and homogeneity. The resultant effects of warming treatments in the different populations and salinity treatments were compared by performing Kruskal–Wallis test using Statistica Software (Statasoft, Tulsa, OK, USA). Significant and highly significant values were assumed when the probability value (*p*-value) was smaller than 0.05 and 0.01 respectively. Multivariate analysis was also conducted using Primer 6 software [67]. A Canonical Analysis of Principal Components (CAP) was also performed using the physiological traits as inputs, to test the efficiency of the variables in describing the populations’ behavior under altered thermal environments, but also to analyze this behavior, producing a statistically tested canonical plot. To evaluate the changes in photochemical and fatty acid metabolism as a whole, a multivariate approach was applied [46]. Canonical analysis of principle (CAP) coordinates, using Euclidean distances, were used to visualize differences in multivariate space regarding studied photochemical variables and fatty acid relative composition, as well as to determine the allocation efficiency to different treatment groups. This multivariate approach is insensitive to heterogeneous data and frequently used to compare different sample groups using the intrinsic characteristics of each group (metabolic characteristics) [30,46,68]. Multivariate statistical analyses were conducted using Primer 6 software [67].

## 5. Conclusions

This study provides new insights on the relationship between environmental history and tolerance variation of *Spartina patens* to salinity. Biophysical and biochemical intraspecific data variation suggests that heavy metal pre-conditioning has a considerable and significant influence on the salinity tolerance mechanisms and salinity resistance of these plants. When comparing marshes, individuals from the pristine site appear to withstand the harshest photochemical consequences as seen by the decrease of the chlorophyll *a*/*b* ratio, through salt concentrations, opposite to the increasing tendency found in the pre-conditioned *S. patens*. These responses were correlated with the highly significant increase in oleic acid found only in *S. patens* from the contaminated marsh, indicating that these plants have an effective light-harvesting complexes stabilization mechanism. Moreover, individual from the pristine marsh exhibited impairments in the LHC mechanisms, coupled with the comparatively deficient energy dissipation mechanisms at high salinities, seems to lead to higher ROS generation and as a consequence of higher plant damage degree. Therefore, it could be concluded that, as salinity increases, the heavy metal contaminated marsh (i.e., Rosario) may generate a more aggressive *S. patens* invasion and spreading, and consequently a more negative ecological effect in the marsh biodiversity especially at high salinities (800 mM NaCl) where the fitness variation between populations is more significant. Therefore, pre-conditioning history seems to potentially be a key factor in the understanding of intraspecies response to future constraints and, subsequently, essential when considering ecological evolution to climate change realities.

## Figures and Tables

**Table 1:** Fatty acid relative content (%, average ± standard error, N = 5) namely, pentadecanoic acid (C15:0), palmitic acid (C16:0), trans-delta 3-hexadecenoic acid (C16:1t), hexadecatrienoic acid (C16:3), stearic acid (C18:0), oleic acid (C18:1), linoleic acid (C18:2) and linolenic acid (C18:3) in *S. patens* leaves from pristine and heavy metal contaminated sites, along with the tested NaCl concentrations. Letters indicate significant differences between treatments at *p* < 0.05; asterisks mark significant differences between populations at *p* < 0.05.

Salinity (mM)		15:0	16:0		16:1t		16:3	18:0	18:1	18:2	18:3
0	Pristine	4.16 ± 0.42 ^a^	12.12 ± 0.72 ^a^	*	1.34 ± 0.37 ^a^	*	1.13 ± 0.44 ^a^	6.26 ± 1.68 ^a^	0.97 ± 0.38 ^a^	15.55 ± 0.66 ^a^	58.46 ± 3.00 ^a^
	Contaminated	2.82 ± 1.18 ^A^	15.34 ± 1.78 ^A^		0.42 ± 0.24 ^A^		3.46 ± 1.57 ^A^	0.94 ± 0.38 ^A^	0.00 ± 0.00 ^A^	15.64 ± 0.61 ^A^	61.37 ± 3.65 ^A^
400	Pristine	4.07 ± 0.85 ^a^	12.78 ± 0.59 ^a^	*	1.02 ± 0.41 ^a^		0.00 ± 0.00 ^a^	5.35 ± 0.87 ^a^	1.74 ± 0.27 ^a^	15.79 ± 0.96 ^a^	59.21 ± 1.01 ^a^
	Contaminated	3.20 ± 0.47 ^A^	14.92 ± 1.03 ^A^		0.96 ± 0.21 ^A^		5.08 ± 0.98 ^A^	1.71 ± 0.50 ^A^	0.00 ± 0.00 ^A^	16.49 ± 0.71 ^A^	57.65 ± 1.41 ^A^
800	Pristine	4.66 ± 1.20 ^a^	16.03 ± 1.28 ^b^	*	3.84 ± 0.86 ^b^	*	2.47 ± 0.47 ^a^	1.36 ± 0.18 ^a^	0.23 ± 0.29 ^a^	14.06 ± 1.25 ^a^	57.25 ± 1.34 ^a^
	Contaminated	4.01 ± 0.39 ^B^	13.77 ± 0.93 ^A^		1.59 ± 0.37 ^A^		9.00 ± 3.05 ^A^	2.17 ± 0.74 ^A^	0.00 ± 0.00 ^A^	16.41 ± 1.31 ^A^	53.05 ± 2.87 ^A^

**Table 2:** Summary of fluorometric analysis parameters and their description.

JIP-Test	
Rapid Light Curves (RLCs)	
rETR	Relative electron transport rate at each light intensity (rETR = QY × PAR × 0.5).
ETR_max_	Maximum ETR after which photo-inhibition can be observed.
α	Photosynthetic efficiency, obtained from the initial slope of the RLC.
Area	Corresponds to the oxidized quinone pool size available for reduction and is a function of the area above the Kautsky plot.
N	Reaction center turnover rate.
S_M_	Corresponds to the energy needed to close all reaction centers.
M_0_	The net rate of PS II RC closure.
δR_0_	PS I efficiency in reducing its electron acceptors.
P_G_	Grouping probability, directly related to PS II antennae connectivity.
ABS/CS	Absorbed energy flux per cross-section.
TR/CS	Trapped energy flux per cross-section
ET/CS	Electron transport energy flux per cross-section.
DI/CS	Dissipated energy flux per cross-section.
RC/CS	The number of available reaction centers per cross-section.
